# Seasonal change in the wetting characteristics of the cuticle of the Collembola *Cryptopygus clavatus* (Schött, 1893)

**DOI:** 10.1007/s00435-015-0254-y

**Published:** 2015-02-06

**Authors:** Håkon Gundersen, Christian Thaulow, Hans Petter Leinaas

**Affiliations:** 1 Department of Engineering Design and Materials, Norwegian University of Science and Technology, NTNU, Richard Birkelands vei 2b, 7491 Trondheim, Norway; 2 Department of Biosciences, University of Oslo, P.O. Box 1066, Blindern, 0316 Oslo, Norway

**Keywords:** Collembola, Cuticle, Seasonal adaptation, Superhydrophobic, Wetting

## Abstract

The littoral Collembola *Cryptopygus clavatus* spends the summer submerged, grazing on algae under water, and the winter on dry land. The cuticles of Collembola are, in general, highly water repellent, often superhydrophobic; the cuticle of *C. clavatus* has, in contrast, been described as not water repellent. Wetting properties are closely tied to surface structuring, and previous studies of Collembola cuticles have used the pattern of cuticular granules to explain the superhydrophobic properties of these cuticles. The wetting properties of the cuticles of *C. clavatus* were measured on animals acclimated to summer and winter. A significant difference in wetting performance was observed. Animals acclimated to winter conditions showed superhydrophobic non-wetting properties. Animals acclimated to summer conditions were not superhydrophobic, water droplets readily adhered to their cuticles. This large change in wetting behavior of the cuticle could not be explained by changes in the cuticular surface structure, which were very limited. Instead, we suggest a change in the epicuticular wax layer could explain the differences.

## Introduction

The cuticles of Collembola (springtails) are in general highly water repellent, and often superhydrophobic (King et al. 1990; Helbig et al. 2011; Ghiradella and Radigan 1974; Noble-nesbitt 1963; Gundersen et al. 2014). As a result, most Collembola will form a protective plastron and float to the surface upon submersion, and consequently very few Collembola are able to remain active under water (King et al. 1990; Nickerl et al. 2012). In contrast, the cuticle of the littoral Collembola *Cryptopygus clavatus* (Schött, 1893), which spends extensive periods submerged with no visible plastron, has been described as “not water repellent” (Fjellberg 2007). *C. clavatus* grazes on algae in the rocky marine littoral zone. It may be observed creeping along the bottom of brackish rock pools and under the water film on rocks after rain. During winter, *C. clavatus* remains in shelter and does not venture out to graze either on land or in rock pools (Fjellberg 1998, 2011).

**Fig. 1:**
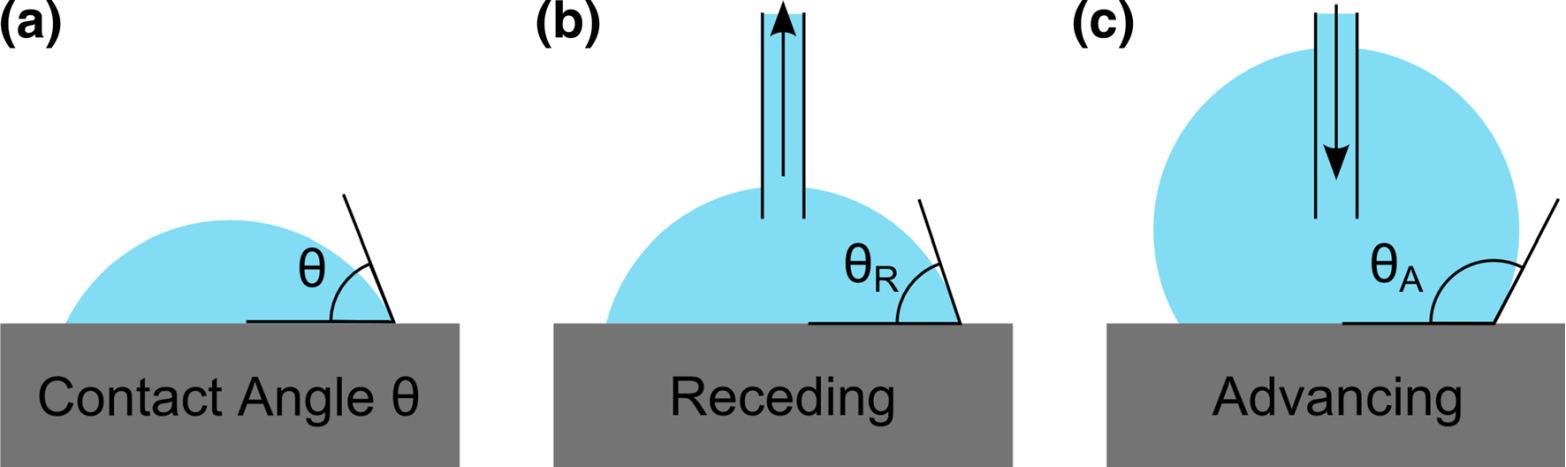
**a** Contact angle $\theta$, the angle between the substrate and the drop. Contact angles of 90° and up are considered hydrophobic. **b** Receding contact angle $\theta _{\text {R}}$ for a shrinking droplet. **c** Advancing contact angle $\theta _{\text {A}}$ for a growing droplet

The wettability of a surface by water is quantified by the contact angle. When a drop of water is resting on a solid surface, it forms a spherical cap (Quéré and Reyssat 2008; Gao and Mccarthy 2009). The relative wettability of the solid results in a contact angle ($\theta$) which can range from 0° (perfectly wetting) to 180° (perfect non-wetting), see Fig. 1. Surfaces with contact angles of 90° and up are referred to as hydrophobic (Gao and Mccarthy 2009; Gu 2002).

If water is carefully siphoned from a drop resting on a solid, the drop will retain the same contact area but change shape until a certain point when the contact line starts receding. Likewise, a drop to which more water is added will retain the same contact area but change shape until the contact line starts advancing (Gao and McCarthy 2006). Any system therefore displays not one, but two defined contact angles, the *receding contact angle* ($\theta _{\text {R}}$) and the *advancing contact angle* ($\theta _{\text {A}}$), see Fig. 1. The difference between these two angles is referred to as the *contact angle hysteresis* ($\Delta \theta$). The mobility of a drop on a surface is determined by the contact angle hysteresis, a low contact angle hysteresis allows drops to move freely on a surface (Gao and Mccarthy 2009; Shirtcliffe et al. 2010). For a surface to be considered superhydrophobic, an advancing contact angle ($\theta _{\text {A}}$) of at least 150° and a contact angle hysteresis ($\Delta \theta$) of no more than 10° is required (Bhushan and Jung 2011).

The cuticles of Collembola are, with few exceptions, superhydrophobic (King et al. 1990; Helbig et al. 2011; Gundersen et al. 2014; Hobæk et al. 2011). Superhydrophobic cuticles in Collembola and other arthropods protect against drowning and pathogens, this occurs through the formation of a plastron upon submersion and through the self-cleaning effect, respectively (King et al. 1990; Gundersen et al. 2014). An animal that respires through the body surface, as Collembola do, or through a tracheal system, as most insects do, can survive indefinitely in aerated water as long as a thin layer of air is retained around the cuticle or the opening of the trachea. This air retention is enabled by superhydrophobic structures on the cuticle, setae, microtrichiae or a combination thereof (Crisp and Thorpe 1948; Bush et al. 2007; Neumann and Woermann 2009). Some insects, notably their larvae, and apparently also the Collembola *C. clavatus* respire under water through direct gas exchange with water.

**Fig. 2:**
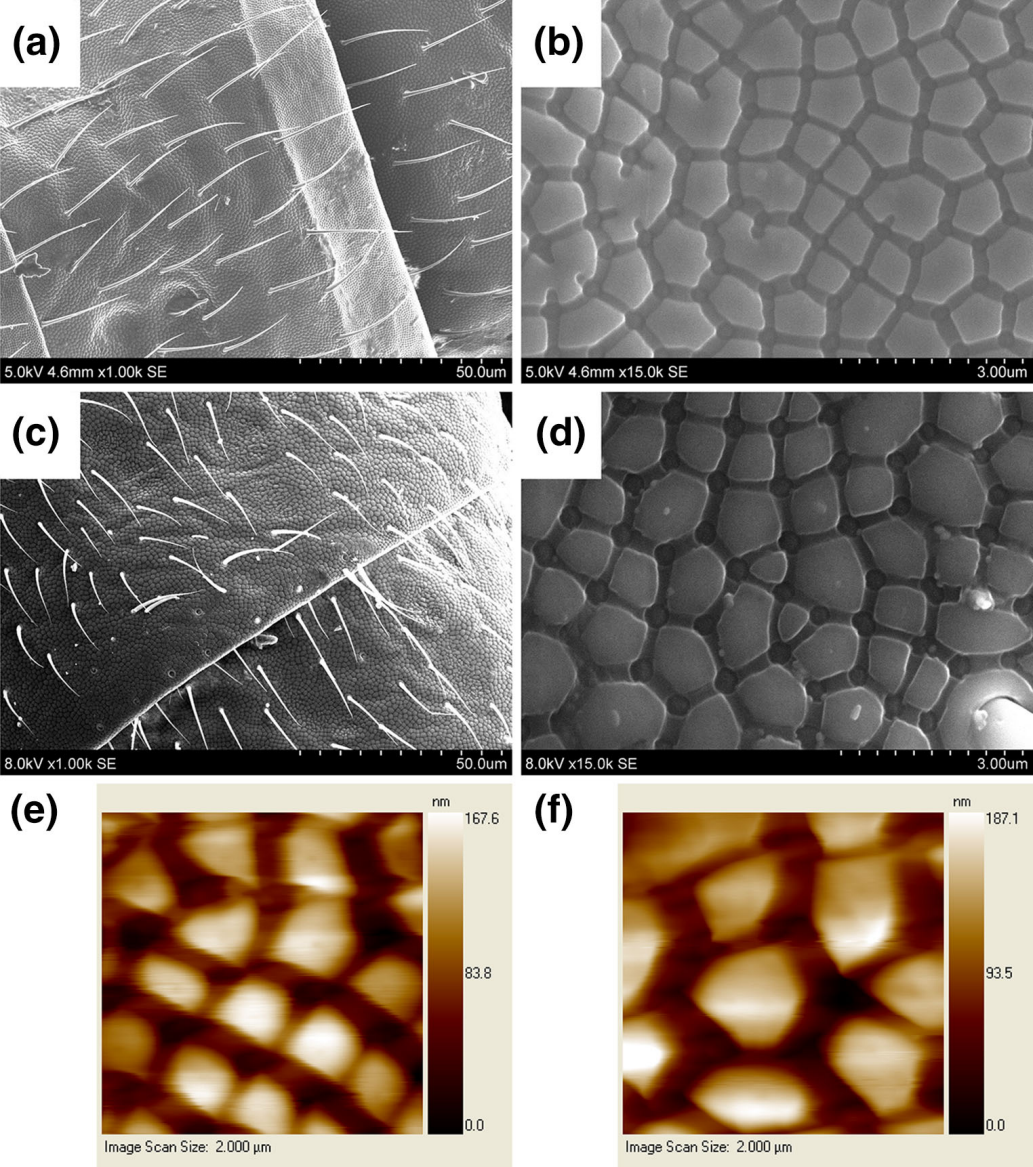
Scanning electron microscopy (SEM) images showing the cuticle of *C.clavatus*. The dorsal metasoma is shown at the joint between the third and fourth segment of summer-acclimated (**a**, **b**) and winter-acclimated animals (**c**, **d**); at 1,000× and 15,000× magnification. Atomic force microscopy (AFM) images are also shown of summer- (**e**) and winter-acclimated animals (**f**), color coding in AFM images are based on height difference

Studies on the wetting behavior of Collembola cuticles have so far focused on the surface structures present on the cuticles (Helbig et al. 2011; Gundersen et al. 2014; Nickerl et al. 2012), which include a network of granules and ridges on a submicron scale (Lawrence and Massoud 1973), see Fig. 2. The presence of overhanging structures (Helbig et al. 2011) and their exact size, distance and height (Gundersen et al. 2014) are likely important factors in determining the wetting behavior of the cuticles. Micro-sized structures with overhang, where the top of the structures are wider than the base, resulting in a reentrant cross section similar to that of a mushroom or one-legged table, are known to enable robust superhydrophobic behavior. Studies of ordered arrays of micropillars with overhanging features (Choi et al. 2009; Cao et al. 2007; Tuteja et al. 2008) and different cross sections (Zheng et al. 2010) have been performed in an effort to create more robust, synthetic superhydrophobic surfaces.

Superhydrophobic effects are known to be a combination of surface chemistry and surface structures, mostly inherently hydrophobic surfaces with a nano-scale or hierarchic surface roughness (Shirtcliffe et al. 2010; Bhushan and Jung 2011). The surface chemistry of Collembola cuticles is modified by epicuticular waxes, which likely play a dual role of protecting against desiccation and improving non-wetting properties (Ghiradella and Radigan 1974; Noble-nesbitt 1963; Gundersen et al. 2014).

The purpose of this work was to study and explain the fundamental change in wetting properties of the cuticle of *C. clavatus* from summer to winter conditions. The cuticle changes from readily wettable in summer conditions to superhydrophobic in winter conditions. Conventional models of wetting and the current understanding of why the cuticles of most Collembola are superhydrophobic would indicate a structural change in the cuticle from season to season, whereas the current understanding of Collembola cuticular structures is that there is no mechanism that can change these during the life span of individual Collembola.

## Experimental method

Live *C. clavatus* (Collembola are non-regulated invertebrates which are not subjected to any special laws or regulations related to animal experiments in Norway.) were collected by Arne Fjellberg at Tjøme, Norway, in October 2011. One set of animals was acclimated to winter conditions by being kept at 3 °C in dry conditions, while another set of animals was acclimated to summer conditions by being kept submerged at 10 °C. The animals were killed with chloroform vapor immediately preceding the experiments.

The wetting properties of the cuticle were determined through the sessile drop method. Drops were applied with a syringe (31 gauge stainless steel, Hamilton Company) and observed with a KSV CAM 200 contact angle goniometer. The data were analyzed with KSV CAM Optical Contact Angle and Pendant Drop Surface Tension Software v.4.04. The test liquid used was de-ionized micropore water. The tests were performed at room temperature at ambient humidity and pressure. Advancing and receding contact angles were attained by leaving the tip of the needle in the drop and adding or siphoning liquid. Drops were deposited on the dorsal metasoma. Some animals were also completely submerged by depositing drops that were larger than the animals. Submerged animals were investigated for visible signs of a retained air layer.

Atomic force microscopy (AFM) was performed in ambient conditions. A TI-750 UBI nanomechanical testing system from Hysitron was used with a 90° cube corner tip to scan the surface in contact mode. AFM brings a piezocontrolled probe with a very fine diamond tip into contact with the sample and allows the scanning of surface images with height data; this method allowed a direct measurement of granule height.

Scanning electron microscopy (SEM) was performed with a Hitachi SU 6600 using the secondary electron detector. Animals were killed and immediately freeze-dried. Dried animals were coated with carbon in an SEM Turbo Carbon Coater from Agar Scientific (typical settings: *t* = 2 × 8 s, *E* = 4.8 kV), prior to the SEM study.

Animals studied in Cryo-SEM were frozen in nitrogen slush and transferred to a Gatan Alto cryo box set to −95 °C; the animals were sputter-coated with approximately 5 nm gold palladium alloy and subsequently transferred to the chamber of a Hitachi S-4800 field emission SEM which was kept at −150 °C. SEM and Cryo-SEM allowed imaging and subsequent measurement of the size and shape of cuticular granules.

Cross sections were milled with a FEI Helios Nanolab dual-beam Focused Ion Beam (FIB) and imaged with the SEM beam of the same tool. Animals were prepared as for SEM imaging with an additional coat of platinum applied with an Edwards Sputter Coater S150B (typical settings: *t* = 60 s, *I* = 20 mA). A thicker protective field of platinum was applied with the FIB system on the area where the cross section was performed. FIB enables site-specific milling with nanometer resolution, which allowed cross sections of granules to be obtained.

## Results

**Fig. 3:**
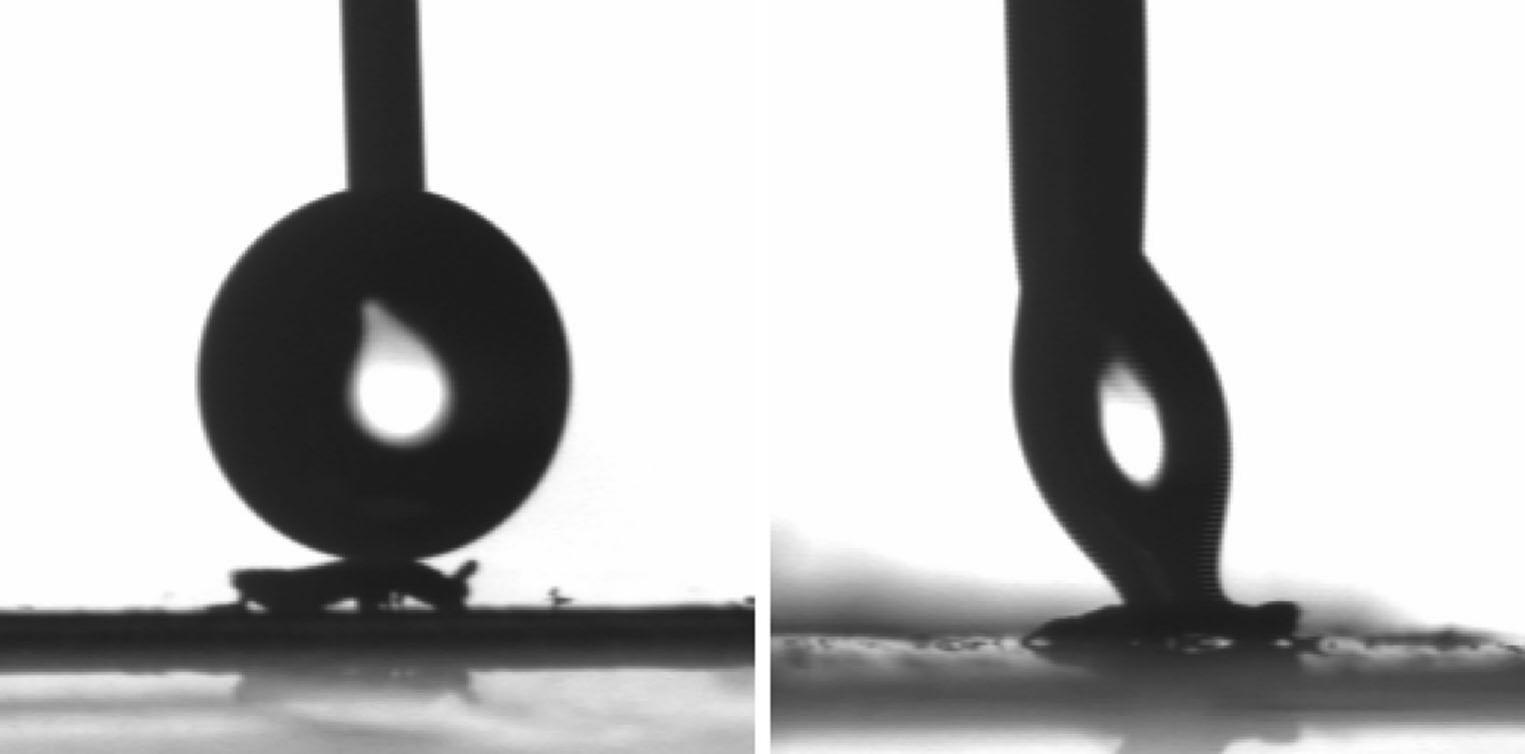
Images from the contact angle goniometer illustrating highly contrasting behavior with water forming a round droplet on a winter-acclimated animal and adhering to a summer-acclimated animal

Contact angles are presented in Table 1: the results of the summer-acclimated animals are the same as those previously reported in a wider study of the wetting of Collembola cuticles in a large selection of species (Gundersen et al. 2014). When subjected to the commonly used criteria for superhydrophobic surfaces (Guo et al. 2011), an advancing contact angle ($\theta _\text {A}$) of at least 150° and a contact angle hysteresis ($\Delta \theta$) of no more than 10°, the cuticles of specimens acclimated to winter conditions were found to be superhydrophobic, while specimens acclimated to summer conditions were not superhydrophobic. Test droplets were observed to cling to summer-acclimated animals, while they were deflected by the winter-acclimated animals, see Fig. 3.

**Table 1:** Contact angles: advancing contact angles ($\theta _{\text {A}}$), receding contact angles ($\theta _{\text {R}}$), an contact angle hysteresis ($\Delta \theta$) of winter- and summer-acclimated animals; all values are given with a statistically attained standard deviation value from a set of measurements

	*θ*_A_	*θ*_R_	$\Delta \theta$	Superhydrophobic
Winter-acclimated	166° ± 2°	166° ± 2°	≈0° ± 3°	Yes
Summer-acclimated	140° ± 3°	118° ± 11°	22° ± 11°	No

**Fig. 4:**
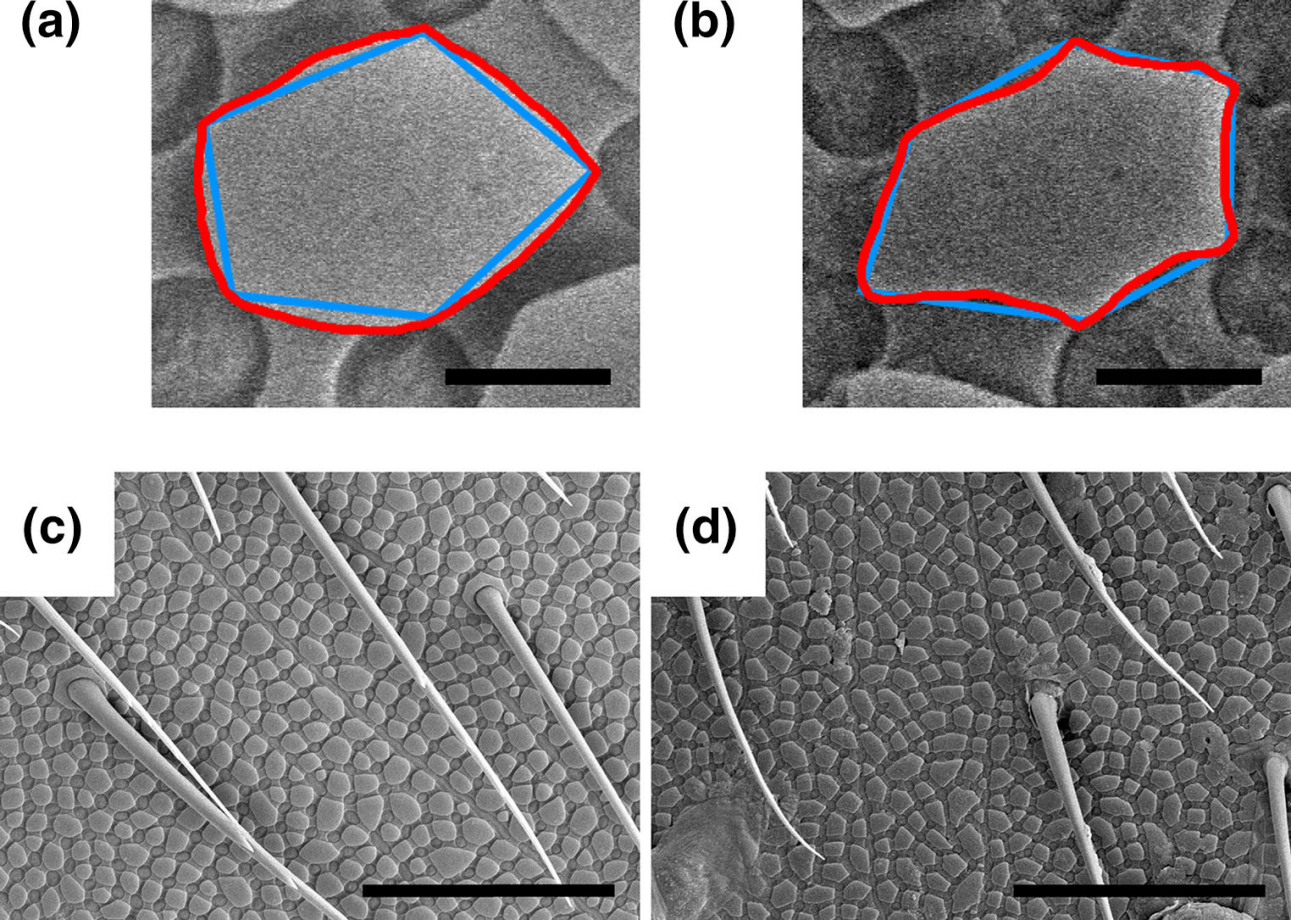
Differences in the cuticle granule curvature for animals acclimated to winter (*left*) and to summer (*right*). Winter-acclimated animals tend toward convex curvature, as compared to straight-edged granules. Summer-acclimated animals tend toward concave curvature, as compared to straight-edged granules. **a**, **b** Granules traced in *red*, with straight-edged equivalent granules indicated in *blue*. **c**, **d** The general cuticle structure.* Scale bars* are 500 nm (**a**, **b**) and 10 μm (**c**, **d**)

Figure 2a, c shows the cuticle at 1,000× magnification with the joint between the third and fourth segment of the dorsal metasoma visible. Figure 2b, d shows the cuticle of the same region at 15,000× magnification. Figure 2e, f shows AFM images of the cuticular structure. The SEM and AFM images were used to determine the surface structure characteristics used in the common wetting models, i.e., the pitch, size, and shape of granules, and these are presented in Table 2, see (Gundersen et al. 2014) for a close discussion on the determination of such characteristics from a SEM image. The sole significant difference found was a change in the curvature in the edges of the granules, which in winter-acclimated animals were straight to convex and in summer-acclimated animals tended to be straight to concave (see Fig. 4). This difference can be quantified by comparing the area contained within a traced circumference around a granule with the area contained within an equivalent straight-edged granule. The ratio between these areas (traced in red and blue in Fig. 4) was $\approx 1.02 \pm 0.02$ for summer-acclimated animals and $\approx 1.14 \pm 0.02$ for winter-acclimated animals.

**Table 2:** Surface structure characteristics: distance between granules (*d*_0_), height of granules (*H*), length of the three-phase contact line for the wetting system of one granule (*L*), the nominal area of the air pocket between granules (*A*), the nominal surface area of a granule (*A*_s_)

	*d*_0_	*H* (μm)	*L* (μm)	*A* (μm)	*A*_s_
Winter-acclimated	0.42 ± 0.06	0.12 ± 0.01	2.2 ± 0.8	0.65 ± 0.5	0.39 ± 0.3
Summer-acclimated	0.38 ± 0.06	0.12 ± 0.01	2.4 ± 0.4	0.73 ± 0.2	0.42 ± 0.1

**Fig. 5:**
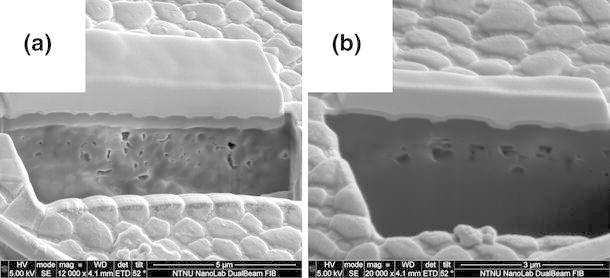
Scanning electron microscope (SEM) images of cross sections of cuticles milled with a Focused Ion Beam (FIB). Images with 12,000X and 20,000X show a cross section of the granular structure covering the dorsal metasoma of a winter-acclimated animal (**a**) and a summer-acclimated animal (**b**). There are no indications of overhanging structures on the granules, which appear quite flat

Cross sections of the cuticle obtained with dual-beam Focused Ion Beam (FIB) milling and SEM imaging are presented in Fig. 5a, b. The purpose of imaging cuticle cross sections was to observe the presence or lack of overhanging structures, which fundamentally alters wetting behavior when present. No overhanging geometry was observed in any cross sections.

## Discussion

A significant, qualitative difference in wetting properties was observed for summer-acclimated animals of *C. clavatus* as compared to winter-acclimated animals. The properties for summer-acclimated animals are in stark contrast to the properties observed on most Collembolan cuticles, which are almost universally superhydrophobic (Gundersen et al. 2014; Helbig et al. 2011). A superhydrophobic cuticle would, however, make the usual summer behavior of *C. clavatus* impossible (Fjellberg 1998, 2011). According to the prevailing knowledge of wetting and superhydrophobic surfaces, this change in wetting must be caused by either a change in surface structuring, a change in surface chemistry or a combination of the two (Shirtcliffe et al. 2010; Bhushan and Jung 2011).

The most important surface features on the cuticle of *C. clavatus*, with regards to wetting performance, are the cuticular granules and setae. The cover of setae, while important for the wetting performance for many arthropods (Bush et al. 2007), remained unchanged for summer- and winter-acclimated animals of *C. clavatus*. The granules can be quantified by their size, shape and the distance between them. The size of and distance between the granules remained the same. In terms of shape, the presence or lack of an overhang, or undercut, beneath the granules is an important feature in wetting systems (Tuteja et al. 2008), and has been suggested as the main contributor to the superhydrophobicity of Collembola cuticles (Helbig et al. 2011). However, as no overhang was observed on either summer- or winter-acclimated animals, this cannot explain the change in wetting behavior either. The only observable change in surface structure was a slight shift in the shape of the cuticle granule edges from convex curvature on winter-acclimated animals to concave curvature on summer-acclimated animals. Adjusting for this small change in contact line length and granule area in classical (Cassie and Baxter 1944) and modified wetting models (Zheng et al. 2010; Choi et al. 2009) does not yield qualitatively different results. While the physical characteristics of the cuticle surface has been assumed to be the main contributor to the non-wetting properties of Collembola cuticles (Helbig et al. 2011; Gundersen et al. 2014), for *C. clavatus* this is clearly not the case, as evident by the drastic difference in wetting behavior for a very similar surface structure. However, the minor change in granule shape could reflect changes in the wax layer.

The thicker parts of the cuticle surface are covered by an epicuticular wax layer that blocks gas exchange (Noble-nesbitt 1963). Covering the cuticle in a wax layer is a trade-off between efficient respiration and protection against desiccation, as Collembola respire, but also lose water by evaporation, through the gas permeable sections of the cuticle that lack a wax layer (King et al. 1990; Noble-nesbitt 1963; Leinaas and Fjellberg 1985). The distribution of permeable surface area and gas blocking areas form the well-known cuticular patterns of Collembola (Lawrence and Massoud 1973; Leinaas and Fjellberg 1985). This has the added effect of creating the superhydrophobic effect which is present in many Collembola (Helbig et al. 2011; Noble-nesbitt 1963; Gundersen et al. 2014) as the wax layer provides the hydrophobic surface chemistry which, in combination with micro- or sub-micro-scale roughness, is required for superhydrophobic non-wetting (Gao and McCarthy 2006; Shirtcliffe et al. 2010; Bhushan and Jung 2011). A reduction in the epicuticular wax layer as part of the summer acclimation can therefore explain the change in wetting behavior.

The cover of epicuticular waxes is subjected to two different selections: one which balances respiration and desiccation and one in which a superhydrophobic cuticle is the result. Modification of the cuticle structure as adaptation to specific drought exposure of the habitat has been well documented (Leinaas and Fjellberg 1985). By comparison, evolution of superhydrophobicity is less obvious, as it seems to be a general characteristic of Collembola from different habitat types, and not limited to habitats that are especially exposed to flooding (Gundersen et al. 2014). However, the seasonal change in the wetting properties of *C. clavatus* clearly shows the result of a direct selective change. While superhydrophobic cuticles protect Collembola from drowning during submersion (King et al. 1990; Ghiradella and Radigan 1974), it would also prevent *C. clavatus* from its characteristic underwater grazing behavior. Plastrons (a local bubble of retained air) around superhydrophobic cuticles provide enough buoyancy to prevent the submersion of animals during normal conditions, or return the animal to the surface after forced submersion. *C. clavatus* would not be able to overcome the buoyancy of a plastron nor would it be able to graze and walk underwater with a plastron. During winter, there is no need for the ability to graze under water, as *C. clavatus* spends the season in inactivity. However, the need to prevent water loss is ever-present, as is the ability to survive occasional events of submersion during flooding.

It is therefore likely that the change in wetting behavior is an adaption to a twofold challenge. The non-wetting effect needs to be reduced in summer to allow grazing, while the water vapor permittivity needs to be reduced in winter. Since both of these qualities may be controlled by the extent and chemical characteristics of the epicuticular wax layer, a seasonal change in the wax layer can explain both how *C. clavatus* is able to adapt, as well as why it shows such excellent water repellence in winter. A change in the epicuticular wax layer could also explain the small change in granule shape that was observed. A more extensive wax cover on the top of the granules in animals acclimated for winter could result in the more convex shape.

The changes in the wetting properties of the cuticle of summer- and winter-acclimated animals of *C. clavatus* cannot be explained by structural changes of the cuticle. There is no known mechanism that would allow individual Collembola to change the shape and size of its granules; and no change was observed. The changes in wetting properties can, however, be explained by changes in the epicuticular wax layer, and such changes are already a known mechanism of adaptation to drought exposure in other species of Collembola. We therefore suggest changes in the epicuticular wax layer as a model of explanation for the seasonal changes in wetting properties. We stress that more detailed studies of the cuticular waxes of *C. clavatus* and other Collembola are needed if a complete understanding of their wetting, and changes in wetting performance is to be reached. This would contribute to the understanding of natural superhydrophobic surfaces, where surfaces with switchable wetting properties are especially interesting, and would also be of great interest to the evolutionary understanding of how these animals adapt to their environments.
